# Biopolymer-Based Electrospun Nanofibers for Wound Healing, Regeneration, and Therapeutics

**DOI:** 10.3390/ma19071443

**Published:** 2026-04-03

**Authors:** Ashok Vaseashta, Sedef Salel, Nimet Bölgen

**Affiliations:** 1 Strategic Research, International Clean Water Institute, Manassas, VA 20108, USA; 2 Research Institute, University of Bucharest, Șoseaua Panduri 90, 050663 Bucharest, Romania; 3 Chemical Engineering Department, Engineering Faculty, Mersin University, Mersin 33343, Türkiye; sedefsalel@gmail.com (S.S.); nimetbolgen@yahoo.com (N.B.)

**Keywords:** electrospun nanofibers, wound dressing, wearable devices, biopolymers, stimuli-responsive dressings, biocompatibility

## Abstract

The management of complex acute and chronic wounds remains a formidable challenge in modern medicine, underscoring the urgent need for advanced therapeutic strategies that accelerate healing, prevent infection, and promote functional tissue regeneration. Electrospun nanofibers have attracted considerable attention in the biomedical field due to their extracellular matrix-like architecture, high surface area, interconnected porosity, and tunable physicochemical composition, which drive advances in wound regeneration, tissue engineering, and biopolymer-based therapeutics. In wound healing, nanofibrous dressings composed of natural polymers such as chitosan, gelatin, collagen, and cellulose promote cell attachment and proliferation, support angiogenesis, and enable infection control while delivering bioactive agents, thereby addressing significant challenges related to inflammation, biocompatibility, and antimicrobial resistance. In tissue engineering, aligned and hierarchically organized scaffolds fabricated from biopolymers such as collagen, gelatin, chitosan, and cellulose enhance the guided orientation of cells, differentiation, and functional regeneration of neural, musculoskeletal, vascular, and skin tissues. In addition to their conventional regenerative applications, recent studies have demonstrated that electrospun biopolymer nanofibers can be used in multifunctional biomedical platforms, including smart and stimuli-responsive systems for drug delivery, biosensing, regenerative interfaces, and wearable medical technologies. The integrated constructs that incorporate diagnostic or therapeutic functionalities, hybrid fabrication approaches that combine 3D printing with electrospinning, and intelligent biopolymer frameworks that enable telemedicine, real-time physiological monitoring, and personalized regenerative therapies offer new opportunities for developing improved biomedical systems. Overall, these advances position electrospun nanofiber systems as promising biomaterials for next-generation biomedical innovation. This review summarizes recent progress in tissue-engineered scaffolds, wound dressings, fabrication strategies for integrative therapeutics, and wearable devices with transformative potential for biomedical applications. Finally, the review addresses significant challenges related to scalability and clinical translation. It offers perspectives on future directions, including the integration of artificial intelligence and the regeneration of complex skin appendages, which will shape the next generation of nanofiber-based wound-healing therapies.

## 1. Introduction

Biomaterials that can support the regeneration of tissue, the healing of wounds, and biomedical functionalities are one of the most important components in regenerative medicine and biomedical engineering applications [1]. For this purpose, biopolymer-based materials have attracted significant attention because of their biocompatibility, biodegradability, and resemblance to native extracellular matrix (ECM) components [2,3]. Another very important component for tissue engineering is the scaffolds that can combine these biopolymers with bioactive agents and provide the necessary structural properties [4]. Electrospinning has arisen as a powerful fabrication method to process biopolymers into effective nanofibers that resemble natural ECM with high porosity, high surface area, and an interconnected structure [5,6]. Biopolymer-based electrospun nanofibers offer high cell adhesion, migration, and proliferation, together with effective transportation of oxygen, nutrients, and bioactive agents [7,8]. These peculiar properties make biopolymer-based nanofibers versatile systems for tissue engineering fields such as neural, musculoskeletal, and vascular tissue engineering, as well as wound healing [9,10]. Some of the most commonly used biomaterials that are used for these applications could be given as chitosan, collagen, gelatin, and cellulose [11,12,13,14].

Beyond healing and regeneration, recent studies have shown that biopolymer-based electrospun nanofibers can be used in smart, multifunctional biomedical platforms, including stimuli-responsive drug delivery systems, biosensors, wearable nanofiber devices, and theranostics. Stimuli-responsive drug delivery systems have been designed to respond to internal and external stimuli for targeted and controlled drug release; internal stimuli include changes in pH, enzymatic activities, or reactive oxygen species (ROS), while external stimuli include light, temperature, and magnetic fields [15,16]. On the other hand, biosensors that use nanofibers benefit from their high surface area and controllable surface chemistry to acquire more sensitive detections for biomolecules, pathogens, and physiological cues [17,18,19]. Wearable healthcare devices further advance their capability to track physiological parameters such as heart rate, respiration, motion, temperature, and sweat compositions by integrating electrospun nanofibers for increased flexibility, breathability, and skin biocompatibility [20,21]. These wearable nanofiber systems can allow personalized healthcare tracking in daily life and can also be integrated in digital platforms for connection with physicians, especially in case of health-related emergencies [22,23]. Moreover, theranostic nanofiber systems combine therapeutic approaches with diagnostic techniques, especially for cancer treatments and wound infections, for which real-time monitoring of the treatment is highly important [24,25,26]. Collectively, these platforms indicate that electrospun biopolymer nanofibers, as versatile materials, can increase the effectiveness of smart and multifunctional biomedical applications.

The geometric structure and mechanical properties of electrospun biopolymer-based nanofibers can be more precisely controlled and enhanced by combining electrospinning with 3D printing techniques [27]. This hybrid manufacturing method can allow increased mechanical stability and biofunctionality [28]. Despite the many advantages of electrospun nanofibers, challenges related to large-scale manufacturing, biopolymer stability, and clinical translation should be considered [29,30]. In this review, we highlight recent progress in biopolymer-based electrospun nanofibers for regenerative medicine, wound-healing applications, smart stimuli-responsive drug delivery systems, biosensors, wearable technologies, theranostics, and hybrid fabrication strategies; we then briefly discuss challenges and future perspectives for developing clinically applicable, multifunctional biopolymer nanofiber-based biomedical platforms.

## 2. Fundamentals of Electrospinning

**Figure 1:**
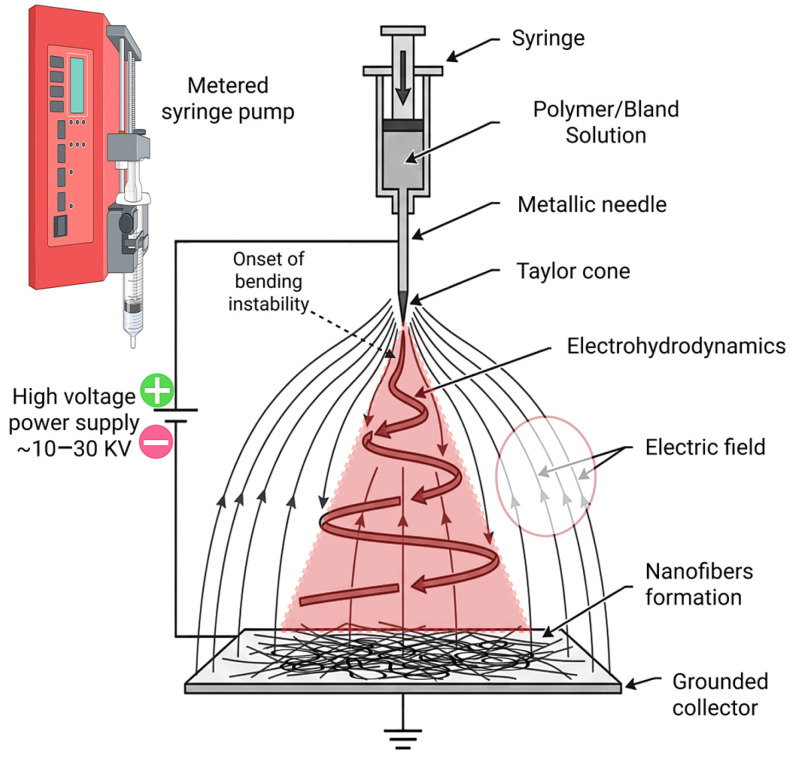
Schematic representation of an electrospinning setup.

Electrospinning is a well-established method to produce fine nanofibers from natural or synthetic polymers. The electrospinning setup includes a syringe, a syringe pump, a collector, and a high-voltage supply. In the process, a syringe is loaded with a polymer solution and placed in the syringe pump [31,32]. High voltage is applied between the polymer-loaded syringe and the collector, causing a polymer jet to emerge toward the collector. The solvent of the polymer solution is evaporated in this process, and polymer fibers are collected on the collector surface [33,34]. A schematic of the electrospinning setup is shown in Figure 1. Fiber structures, mechanical properties, and surface chemistry are affected by process parameters such as applied voltage, concentration of the polymer, flow rate of the polymer, and syringe tip to collector distance [35]. So, the nanofibers can be engineered to exhibit many necessary properties for imitating natural extracellular matrix (ECM), such as high surface area, high porosity, and adjustable fiber diameter [36]. While electrospun scaffolds mimic the natural ECM, their porosity and pore size are critical for cell spreading, migration, and nutrient and waste exchange. The efficiency of mass transport is primarily governed by fiber packing density and pore size distribution. Limitations in electrospun scaffolds are associated with high-density packing of fibers and its consequences on cell infiltration [37]. High packing density reduces pore size and limits cell infiltration. By modulating fiber diameter and packing density, cell viability, proliferation, and infiltration can be enhanced. Soliman et al. produced sparse or highly dense PCL mats by electrospinning and demonstrated that low packing density resulted in improved cell viability and proliferation compared to tightly packed scaffolds [38]. Optimizing the balance between fiber diameter and packing density is essential to maintain sufficient nutrient transfer and ensure long-term cell viability in tissue-engineered constructs. These properties are significantly important for many biomedical applications, such as drug delivery, wound healing, and many other tissue engineering applications [39,40].

Along with electrospinning properties, the physicochemical properties of the spinning solution (typically a polymer solution) strongly influence fiber properties. The main physicochemical properties that can affect fiber formation include molecular weight, solubility, viscosity, and electrical conductivity [41]. Natural polymers such as gelatin, collagen, and chitosan can have strong intermolecular hydrogen bonds and polarity, which can prevent stable jet formation in electrospinning [42]. For this reason, natural polymers can often be mixed with synthetic polymers such as poly(ε-caprolactone) (PCL) or poly(vinyl alcohol) (PVA) [43]. Another important factor in electrospinning is the choice of solvent for solubilizing the polymer. The solvent features, such as dielectric constant, volatility, and surface tension, can highly influence jet stability and nanofiber morphology [44]. Furthermore, an optimal level of polymer chain entanglement is crucial for obtaining continuous and uniform nanofibers, as low levels of entanglement, which lead to bead formations or spindle-like structures, can be seen at low polymer concentrations [45]. Moreover, crosslinking is often performed after electrospinning to improve the mechanical properties of nanofibers, especially for natural polymers such as gelatin [46,47].

Electrospun nanofibers fabricated from natural polymers such as collagen and gelatin exhibit limited mechanical stability and rapid dissolution in aqueous media or physiological environments. Therefore, various crosslinking methods are applied to enhance structural integrity and maintain fiber morphology under hydrated conditions. Chemical crosslinking methods, including glutaraldehyde, carbodiimide-based systems, and genipin, have been widely used to form covalent bonding between polymer chains, thereby improving tensile strength and hydrolytic stability. In addition to chemical methods, physical crosslinking approaches such as UV irradiation, electron-beam treatment, and dehydrothermal processing have been used as alternatives to reduce the potential cytotoxicity associated with chemical crosslinkers [48,49]. However, the selection of the crosslinking method must balance mechanical reinforcement and biological functionality. While crosslinking can significantly enhance mechanical stability and reduce degradation rates, it may potentially affect interactions between cells and scaffolds. Therefore, optimizing the crosslinking ratio and using biocompatible crosslinking agents are critical to provide a balance between mechanical stability and biological activity in electrospun scaffolds designed for biomedical applications [50].

Along with the conventional electrospinning, many composite electrospinning methods have been developed to further improve the functionality of nanofiber scaffolds for biomedical applications. Some of these methods could be given as coaxial electrospinning, emulsion electrospinning, side-by-side electrospinning, and blend electrospinning, and they can allow the fabrication of more complex fiber structures with multiple components [51]. For instance, coaxial electrospinning can allow the formation of core–shell nanofibers, which can encapsulate drugs, proteins, or growth factors within the fiber core, and the shell enables a controlled and sustained drug release by acting as a physical barrier between the core and the solution [52]. Moreover, emulsion electrospinning can produce nanofibers that have the ability to integrate both hydrophilic and hydrophobic bioactive agents within polymer matrices by using water-in-oil (W/O) emulsions or oil-in-water (O/W) emulsions, respectively [53]. These composite electrospinning strategies significantly enhance the abilities of electrospun scaffolds by enabling multifunctional systems with improved biological performance, controlled drug delivery, and enhanced healing capabilities [54]. Thus, advanced electrospinning techniques could be promising to develop next-generation nanofiber-based materials for wound healing, regeneration, and therapeutic applications [55].

As electrospinning moves from laboratory research toward industrial use, understanding electrospinning principles is key to controlling and tailoring fiber structures. Optimization of physical and chemical parameters allows precise modification of electrospun materials, making them suitable for applications in smart textiles, wearables, devices, and advanced medical technologies. Combining electrospinning with emerging technologies such as additive manufacturing, 3D/4D printing, and microfluidics enables the fabrication of multifunctional materials and devices with controlled architectures and enhanced therapeutic functions [56]. The effectiveness of these biomedical platforms depends on functional integration and modifying the scaffold structure to produce specific biological or protective responses. An example of such advanced functionalization is the development of water-borne polymer nanocoatings (nanoworms), which can be applied to surfaces to rapidly inactivate viruses, including SARS-CoV-2 [57]. Applying such active, anti-pathogenic nanocoatings with electrospun materials provides promising opportunities for the next generation of wound dressings and advanced personal protective equipment.

## 3. Tissue Engineering Applications of Biopolymer Nanofibers

Tissue engineering benefits from cells, scaffolds, and bioactive agents to restore or replace damaged tissues or organs [58]. For creating new tissue-resembling structures, cells are seeded onto a porous scaffold, which maintains support and serves as a platform that enables controlled drug (and growth factors) release under regulated conditions [59]. Electrospun nanofibers are often chosen as scaffold materials because their porous, interconnected structure resembles the extracellular matrix (ECM), provides mechanical stability, and enables cell attachment, proliferation, and differentiation [60,61]. Furthermore, the variability in biopolymer types allows control over degradation rates and mechanical properties suitable for a specific tissue [62,63]. All these properties make electrospun biopolymer nanofibers appealing to many tissue-engineering fields, such as nerve, musculoskeletal, vascular, and dermal tissue engineering [64]. In this section, we will explore each of these tissue engineering fields separately in terms of biopolymer nanofibers, except for the dermal tissue engineering field. Since electrospun nanofibers play a significant role in wound-healing applications, this topic is discussed in more detail in Section 4.

### 3.1. Neural Tissue Engineering

**Figure 2:**
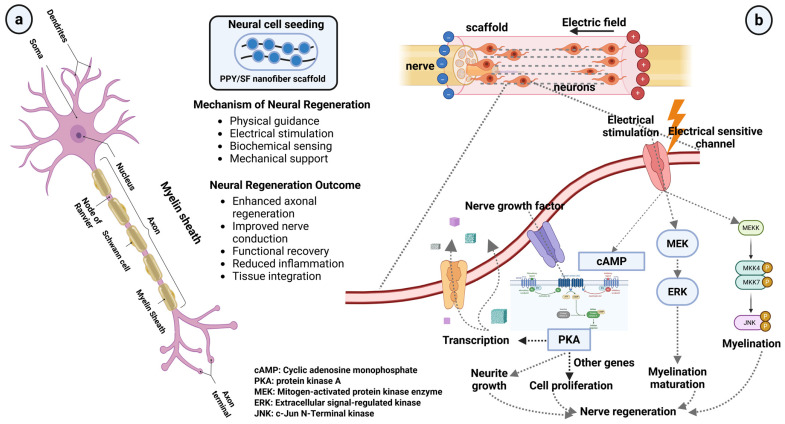
(**a**) Representative neuron structure. (**b**) Schematic showing the use of electrospun Polypyrrole/Silk Fibroin (PPY/SF) nanofiber scaffolds and the possible mechanisms of the promoted neural regeneration.

The human nervous system has very limited regeneration capability. It includes the central nervous system (CNS) and the peripheral nervous system (PNS). Unfortunately, damage to the CNS lacks regenerative solutions, whereas PNS treatments are comparatively simpler. In case of nerve injuries in the PNS, techniques such as surgical reconnection (if the gap in the injury is small) or autografting (for larger gaps) are the conventional ones [65]. However, they have limitations such as multiple surgery requirements, morbidity in donor sites, and poor donor numbers for grafts [66]. The highly complex structure of neurons, as illustrated in Figure 2a, demonstrates the importance of guidance and structural support for effective neural regeneration.

Thus, precisely engineered scaffolds have been suggested as alternate materials for this purpose. Electrospun biopolymer nanofibers are considered promising since they exhibit a high surface area and a similar fibrous structure to the ECM that eases cell attachment, migration, and growth in axons (elongated portions of nerve cells) [67]. Recently, polymeric biomaterials, especially those with high electrical conductivity, have drawn attention for neural regeneration. These include polypyrrole (PPY), polyaniline, polyphenylene, and polythiophene [68,69,70].

Similarly, Zhao and co-workers produced scaffolds from Polypyrrole/Silk Fibroin (PPY/SF) nanofibers, which exhibit high biocompatibility, benefiting from the electrical conductivity of PPY and the mechanical strength of SF (Figure 2b) [71,72] This combined structure demonstrated improvements in Schwann cell alignment, proliferation, and migration, as well as enhanced expression of neurotrophic factors, including Brain-Derived Neurotrophic Factor (BDNF), Nerve Growth Factor (NGF), and Neurotrophin-4/5 (NT-4/5). Neurotrophic factors are known to support neuronal survival, promote axonal growth, and enhance neural regeneration by activating key signaling pathways [73].

Despite these promising studies, electrospun biopolymer nanofibers have both advantages and drawbacks in the field of neural tissue engineering. Their ability to mimic the aligned fibrous architecture of the human ECM can support cell adhesion and migration. In addition, their high surface area and controllable fiber alignments can support the integration of bioactive agents, neurotrophic factors, and conductive materials, which can improve neural signaling and regeneration [74,75]. However, there are many disadvantages worth mentioning. For example, many natural biopolymers have low mechanical stability and rapid degradation rates under physiological conditions, which might decrease the durability of the scaffolds in the long term for neural regeneration [76]. Furthermore, achieving optimal electrical conductivity while maintaining structural stability can be challenging. Another limitation can be given as the adversity of mimicking the high complexity of native neural tissue, which includes vascularization, long-distance neural communication, and functional synapse integration [77]. Thus, further research should focus on these challenges to fully understand the potential of electrospun nanofibers for neural tissue engineering.

### 3.2. Musculoskeletal Tissue Engineering

Musculoskeletal tissues include skeletal muscles, bones, cartilage, ligaments, and tendons. Together, these tissues support the body’s structural integrity, movement, and load-carrying capacity [78]. Damage and disorders in these tissues can reduce self-healing ability, particularly in poorly vascularized tissues such as [79]. Electrospun biopolymer nanofibers can be selected for musculoskeletal tissue regenerations for their capability to mimic the fibrous structures of natural ECM, and for their high porosity, surface area, and controllable fiber designs, which can assist cell adhesion, proliferation, and differentiation [80,81].

In bone tissue engineering, electrospun biopolymer nanofibers produced from collagen, gelatin, chitosan, and silk fibroin have been shown to enhance osteoblast attachment and support osteogenic differentiation, especially when they are combined with inorganic components such as ceramic or minerals like hydroxyapatite, since these materials can improve mechanical strength and provide biochemical signals that stimulate bone matrix deposition and mineralization [82,83,84,85]. For instance, Frohbergh and co-workers developed electrospun chitosan nanofibers that contain hydroxyapatite for bone tissue regeneration and indicated that these scaffolds supported osteoblast-like cell adhesion and proliferation and exhibited better results in osteogenic differentiation markers (e.g., higher alkaline phosphatase (ALP) activity and osteonectin expression) when compared with pure chitosan scaffolds [86].

Ligaments, which connect bone to bone, and tendons, which connect muscle to bone, are predominantly subjected to unidirectional loading, and their ECM mainly consists of type I collagen [87]. Thus, ligament and tendon regeneration can particularly benefit from the highly oriented structure of electrospun nanofibers since it resembles the anisotropic fibrous structure of native collagen fibers [88]. In this context, Yin and co-workers seeded human tendon progenitor cells (hTSPCs) on aligned PLLA fibers, and the cells oriented along the fibers and showed higher levels of tendon-specific gene expression than cells seeded on randomly oriented fibers [89]. A similar mechanism is also observed in skeletal muscle tissue regeneration. The aligned nanofiber structures support myoblast elongations and maturation into multinucleated myotubes [90,91].

Biopolymer nanofibers can offer numerous advantages for musculoskeletal tissue engineering. For instance, their ECM-like structure and adaptable fiber structure can support cell adhesion and proliferation, especially in anisotropic tissues such as tendons, ligaments, and skeletal muscles [92]. Moreover, their high surface-to-volume ratio can allow them to integrate inorganic components such as hydroxyapatite, which can increase osteogenic or myogenic regeneration [93]. Despite promising studies, electrospun biopolymer nanofibers also face challenges in musculoskeletal tissue engineering. For example, natural biopolymers’ limited mechanical strength and rapid degradation behavior under physiological conditions may not always meet the long-term mechanical requirements of load-bearing musculoskeletal tissues [94]. Thus, strategies such as polymer blending and crosslinking are often employed to enhance the structural integrity and functional performance of electrospun scaffolds.

### 3.3. Vascular Tissue Engineering

The main purpose of vascular tissue engineering is to heal or replace damaged blood vessels using cells, bioactive signals, and scaffolds [95]. This field is especially important for small-diameter vascular grafts, as clinical failures can often result from thrombosis (blood clot formation), insufficient endothelialization, compliance mismatch, or intimal hyperplasia (abnormal cell proliferation within the intimal layer of a blood vessel) [96,97]. Electrospun biopolymer nanofibers are attracting attention for vascular tissue engineering applications not only for their ECM-resembling fibrous structure, high surface-to-volume ratio, and tunable porosity, but also for their ability to be fabricated into tubular shapes that resemble blood vessels, with highly controllable wall thicknesses and fiber architectures [98]. Adjusting the properties of tubular nanofibers is crucial to achieving the mechanical and structural features of native blood vessels. A key feature of the blood vessels can be given as the rapid formation of a functional endothelial layer to prevent thrombosis [99]. Therefore, electrospun biopolymer scaffolds should support endothelial cell adhesion and proliferation [100].

Various biopolymers have been studied for the fabrication of vascular graft nanofibers, with emphasis on their biocompatibility, biodegradability, and cell interactions. The most common biopolymers in this field are collagen, silk fibroin, gelatin, and some polysaccharides such as cellulose, chitin, and glycosaminoglycans [101]. Among these biomaterials, silk fibroin attracts particular attention due to its mechanical robustness and slow degradation rate [102,103]. Soffer and colleagues showed that electrospun silk fibroin-based tubular scaffolds exhibited efficient mechanical properties and supported endothelial cell adhesion and proliferation, demonstrating the applicability of silk fibroin for small-diameter vascular graft applications [103]. Furthermore, Liu and co-workers’ study showed that sulfated silk fibroin nanofibers exhibit anticoagulant activity and high cytocompatibility, which are essential properties for biomaterials in contact with blood [104]. Besides silk fibroin, gelatin has been widely used in electrospun vascular grafts. It has been chosen in many studies for integration with these grafts due to its bioactive cell-binding properties, which improve endothelial cell adhesion and proliferation [105]. Gelatin-containing electrospun nanofibers can be used alone or in combination with synthetic polymers, and they generally enhance endothelialization and overall biological performance while maintaining mechanical integrity for vascular applications [106,107].

Despite promising work, several challenges remain to be addressed in the use of electrospun biopolymer nanofibers for vascular tissue engineering. One of the significantly important properties for vascular grafts is long-term hemocompatibility, as inadequate endothelial coverage or poor surface chemistry may cause platelet adhesion and thrombosis [108]. Another challenge could be achieving mechanical properties that can closely resemble the properties of native blood vessels, especially in terms of pressure and compliance [109]. Furthermore, the difficulty in maintaining long-term vascular patency should be considered, because inflammatory responses and intimal hyperplasia may occur after implantation [110]. Thus, recent research primarily focuses on surface functionalization, the incorporation of anticoagulant agents, and advanced scaffold structures that better support blood-material interactions and vascular remodeling.

## 4. Electrospun Biopolymer Nanofibers in Wound Healing Applications

**Figure 3:**
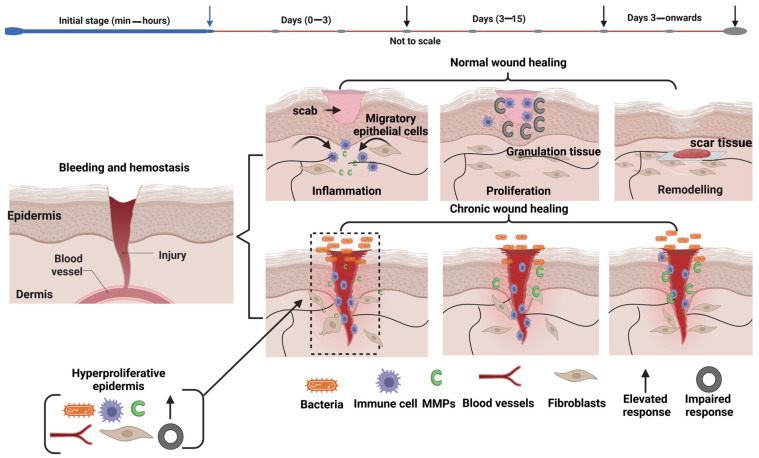
Schematic representation of the wound healing phases and comparison of normal wound healing versus chronic healing.

Wound healing is a dynamic and complex process that includes four main phases: hemostasis, inflammation, proliferation, and remodeling [111]. While in the case of many acute wounds, these phases can be successfully achieved naturally, chronic or infected wounds, or some extreme injuries such as radiation burns, often need external interventions to heal [112,113]. As shown schematically in Figure 3, in normal wounds, healing begins with a phase called hemostasis, during which blood clotting and scab formation stop bleeding. Then, the inflammatory phase (lasts up to 3 days) begins, and immune cells migrate to the wound site to eliminate bacteria and damaged tissue. Afterward, the proliferative phase (lasting up to 12 days) begins, and fibroblasts, endothelial cells, and epithelial cells contribute to tissue regeneration, leading to the formation of granulation tissue. Finally, in the remodeling phase, the tissue matures and a scar forms. In contrast, chronic wounds cannot progress through these stages efficiently and generally remain in a prolonged inflammatory state. Persistent bacterial presence, excessive immune system activity, and elevated matrix metalloproteinase (MMP) levels can harm newly formed ECM, while disrupted fibroblast function and impaired angiogenesis could further delay tissue regeneration. As a result, chronic wounds could have incomplete wound closure. As external wound dressings, electrospun nanofibers are promising candidates due to their ECM-simulating structure, large specific surface area, and high porosity [36]. These properties facilitate oxygen transport, moisture absorption, and the diffusion of nutrients and metabolites to wounded tissues, while supporting cell migration and adhesion for effective wound healing [114,115]. In particular, electrospun biopolymer nanofibers offer unique advantages for wound healing since they can be fabricated from biocompatible, biodegradable, and bioactive materials [116]. For example, natural polymers, including chitosan, gelatin, collagen, cellulose, silk fibroin, alginate, and hyaluronic acid, can contain specific bioactive functional groups that enhance cell adhesion and support the proliferation of key cells such as fibroblasts and keratinocytes during wound healing [117]. Moreover, synthetic polymers, such as PLA, PCV, and PVA are also highly chosen for their controllable mechanical and degradation properties. Table 1 lists comparative properties of the most commonly used biopolymers for electrospun nanofibers for wound healing and tissue regeneration.

Natural biopolymers have been widely used as nanofiber materials, particularly due to their intrinsic bioactivity and favorable interactions with biological tissues. For instance, *Chitosan* nanofibers are known for their antibacterial and hemostatic properties, and their polycationic chemical structure allows electrostatic interactions with negatively charged bacterial cell membranes and supports platelet adhesion and angiogenesis [144,145,146,147]. Similarly, *gelatin* and *collagen* nanofibers can closely mimic native ECM proteins and contain cell-interactive motifs such as Arginine-Glycine-Aspartic Acid (RGD) sequence, which is the most common peptide motif responsible for cell adhesion to the extracellular matrix and for the proliferation of skin cells, such as dermal fibroblasts and keratinocytes [148,149,150,151,152,153,154]. *Cellulose*, the most abundant polysaccharide in nature, although not natively existing in human ECM, can provide excellent physicochemical stability and moisture retention capacity when used as nanofiber materials, and allow effective absorption of wound exudate and maintenance of a moist environment that supports wound healing [155,156,157,158,159,160]. Functional modifications, such as crosslinking, polymer blending, and incorporation of bioactive agents, could further enhance the structural stability and therapeutic potential of these nanofiber scaffolds [46,47,161,162,163]. Beyond these widely studied materials, several additional polymers have expanded the functional landscape of electrospun wound dressings. For example, *Silk fibroin* has been studied because of its high mechanical strength, tunable degradation rates, and long-term mechanical stability, which make it suitable for load bearing or mechanically demanding biomedical applications [102,134]. *Alginate* and *hyaluronic acid* can possess high hydration capacities and immunomodulatory functions that accelerate wound healing and tissue regeneration [136,137]. Furthermore, many electrospinning systems combine natural polymers with synthetic ones such as polylactic acid (PLA) and poly(ε-caprolactone) (PCL) to improve mechanical stability, processability, and controllable degradation rates, and poly(vinyl alcohol) (PVA) is frequently used as a co-spinning agent to facilitate more stable nanofiber formation [127,129,140,143]. Collectively, electrospun nanofibers produced from these biopolymers offer many valuable properties for next-generation wound dressings and can address the complex, multifaceted demands of wound care.

## 5. Smart and Multifunctional Biomedical Platforms

### 5.1. Stimuli-Responsive Smart Drug Delivery Systems

Stimuli-responsive smart drug delivery systems have attracted attention due to their sensitivity to specific environmental changes or external signals and their ability to control the release of bioactive agents [164]. Electrospun nanofibers are commonly used as smart delivery systems because of their large surface-to-volume ratio, high porosity, and ECM-resembling structure. These nanofibers respond to external stimuli by undergoing physicochemical changes such as swelling, linker cleavage, or altered permeability, thereby modulating diffusion rate and release kinetics of bioactive agents. They can provide site-specific, on-demand drug release in response to stimuli in their surrounding environment [165]. The stimuli can be internal or external. While internal stimuli can include variations in pH and elevated levels of Reactive Oxygen Species (ROS), external stimuli can include changes in temperature, light, and magnetic fields [166].

**Figure 4:**
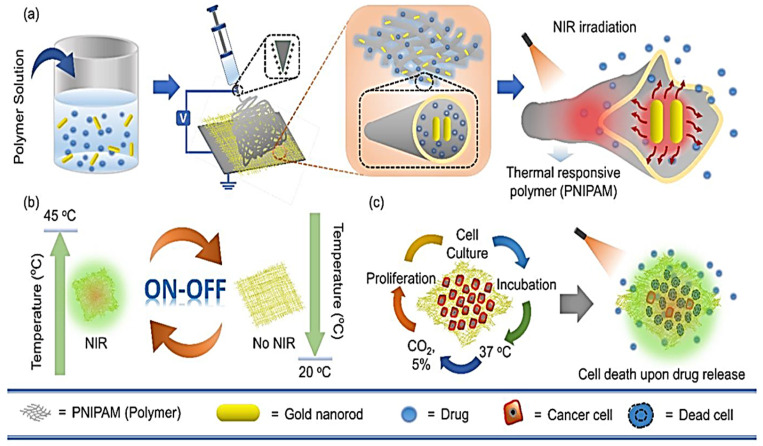
Schematic representation of NIR light-responsive nanofibers containing gold nanorods for on-off drug delivery. (**a**) Fabrication of nanofibers by electrospinning and drug release triggered by NIR. (**b**) Cyclic on-off behavior of nanofibers. (**c**) Cell growth on the nanofibers and cell death following drug release. Reproduced from Ref. [170] under the terms of the Creative Commons Attribution License (CC BY).

Amongst internal stimuli, pH-responsive biopolymer nanofibers have attracted considerable interest, as cancerous or infected wound tissues typically exhibit distinct pH levels. By integrating polyelectrolytes or pH-sensitive polymers into nanofibers, drug release can be effectively targeted to specific sites, thereby minimizing systemic drug exposure [167]. Likewise, ROS-responsive systems exploit elevated oxidative stress at specific sites, which can trigger polymer degradation and drug release from nanofiber systems [168]. Among external stimulus-responsive systems, light-responsive nanofibers, such as near-infrared (NIR)-activated systems, have been widely studied because of NIR light’s deep-tissue penetration and precise targeting capabilities. NIR light activates photothermal agents within the nanofibers, resulting in localized thermal or chemical changes that alter the polymer structure and ultimately enable remotely controlled, spatially precise drug release [169]. For example, Singh and co-workers produced poly(N-isopropylacrylamide) (PNIPAM) nanofibers containing gold nanorods (GNRs) as a drug-delivery system. Upon NIR irradiation, the heat generated by GNRs caused the shrinkage of nanofibers due to the thermal response of the PNIPAM polymer, leading to on-off controlled drug release (Figure 4) [170].

The nanofiber systems can be further improved for precise, controlled drug delivery by being adapted to respond to multiple stimuli, either simultaneously or sequentially [165]. These hybrid systems may be more advantageous in complex microenvironments with multiple pathological signals. Advanced electrospinning techniques, such as coaxial electrospinning and post-fabrication modifications, can allow the creation of nanofibers with the necessary designs for rapid responses to multiple stimuli [171,172]. Consequently, stimuli-responsive electrospun nanofibers represent a versatile and powerful system for smart drug delivery.

### 5.2. Electrospun Nanofiber-Based Biosensors

**Figure 5:**
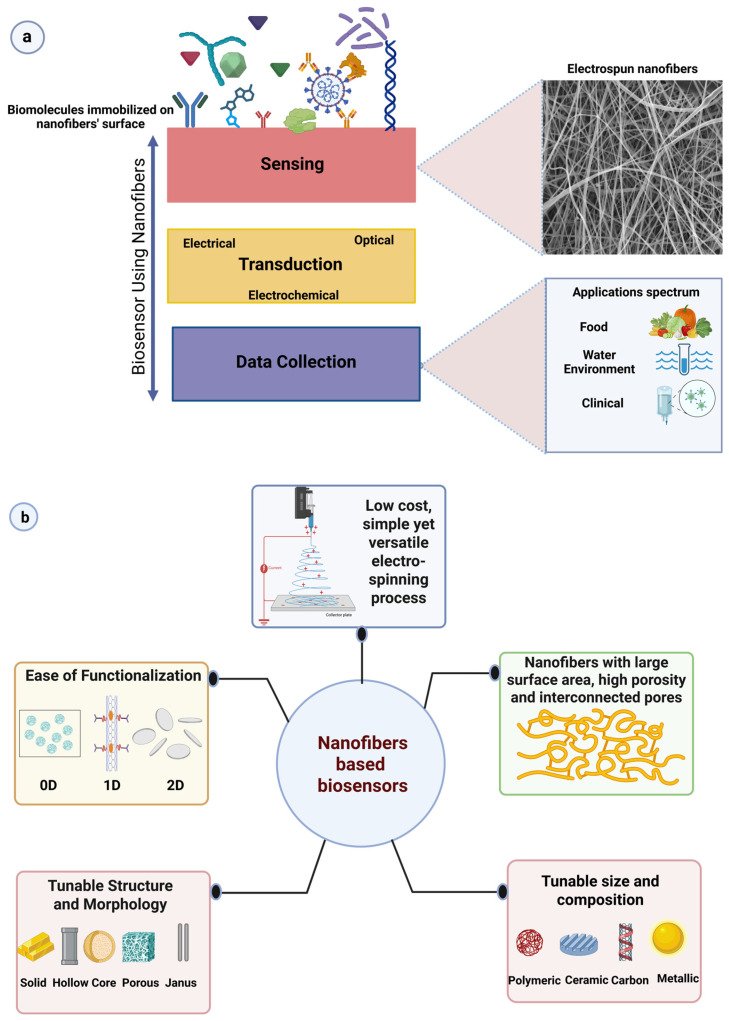
(**a**) Schematic representation of electrospun nanofiber-based biosensors. (**b**) Schematic representation of the advantages of electrospun nanofibers as biosensors.

Sensors are the devices that detect physical, chemical, or biological signals and convert them to quantitatively analyzable outputs [173]. Sensors that integrate biological receptor molecules, such as enzymes, antibodies, and cells, are called biosensors [174]. They have three main components: a sensing layer with a biological recognition element immobilized on its surface, a transducer that converts the biological signal into a measurable signal, and a signal-processing and data-acquisition system that processes and records the signal for analysis (Figure 5a) [175]. The biosensors combine the selectivity of biological recognition elements with the high sensitivity of physicochemical transducers, enabling rapid and specific detection of target analytes [174]. Biosensors are used in various fields, including clinical diagnostics, monitoring, food safety and quality control, and biomedical research [176,177,178]. The effectiveness of a biosensor is strongly linked to the surface area of the sensing layer, since biomarker immobilization and analyte interaction occur within it. Traditional sensor materials can have limited sensitivity due to their low surface area and limited mass transport capacity. However, electrospun nanofiber-based biosensors are highly advantageous for biomolecule immobilization due to their superior surface-to-volume ratio, interconnected, highly porous architecture, ease of functionalization with other nanostructures, low cost, and controllable size and structure (Figure 5b). These properties provide enhanced sensitivity, faster response, and lower detection limits when compared with traditional biosensor structures [179,180].

Electrospun nanofiber-based biosensors can be designed using a wide range of materials to target diverse biomolecules through different transduction strategies [17]. For instance, Yezer and Demirkol developed electrospun nanofibers from cellulose acetate and chitosan on glassy carbon electrodes (GCE), and immobilized glucose oxidase for glucose detection [181]. In another study, Ratlam and co-workers developed a polyaniline (PANi)/carbon quantum dot (CQD)-based electrospun nanofibrous biosensor for dopamine detection, achieving a low detection limit and high selectivity toward neurotransmitters [182]. Furthermore, Fathi and colleagues reported an aptamer-functionalized electrospun carbon nanofiber biosensor capable of detecting Salmonella enterica with a very low detection limit, demonstrating the applicability of nanofiber-based systems for pathogen sensing [183]. Moreover, many studies have shown that integrating conductive compounds, such as carbon nanotubes, graphene, and various conducting polymers, with electrospun nanofibers enhances signal transfer in electrochemical biosensor systems [184,185,186]. Overall, electrospun nanofiber-based biosensors are highly useful sensing systems that integrate materials engineering with advanced biomedical diagnostics and electronics.

### 5.3. Wearable Nanofiber Systems for Real-Time Monitoring and Personalized Healthcare

**Figure 6:**
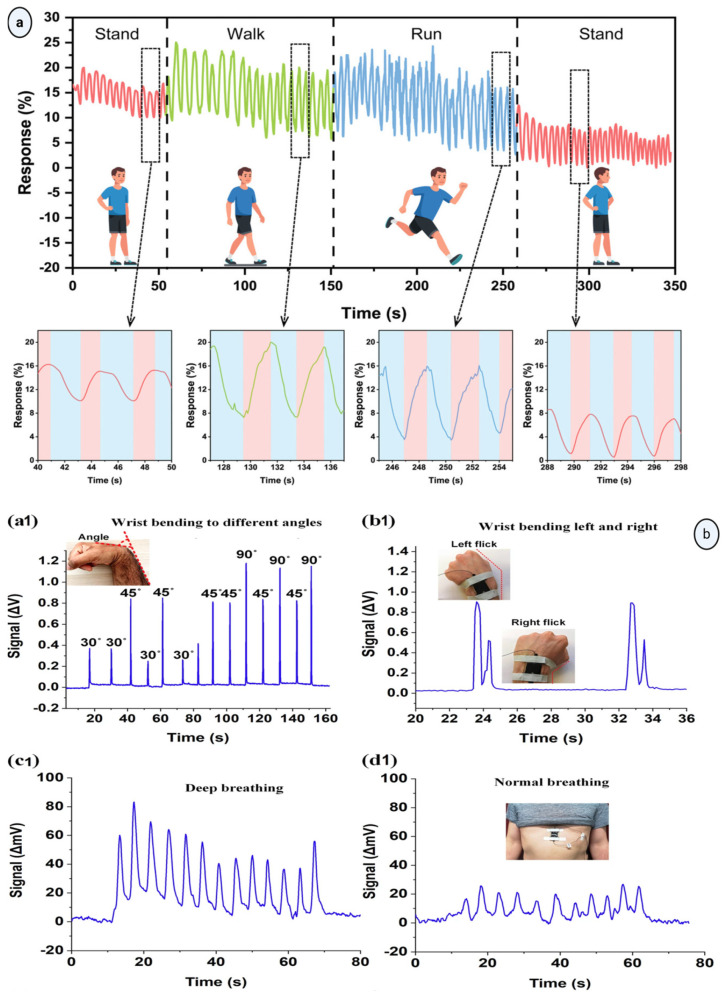
(**a**) Respiration response curves during different motion states. Reproduced from Ref. [193] under the terms of the Creative Commons Attribution License (CC BY). (**b**) (**a1**) Response of the strain sensor to different wrist bending angles; (**b1**) response of the strain sensor to sideways wrist bending; (**c1**) response of the strain sensor to deep and (**d1**) normal breathings, respectively. Reproduced from Ref. [194] under the terms of the Creative Commons Attribution License (CC BY).

Recently, wearable healthcare technologies that aim to capture physiological or biochemical cues in our daily lives and convert them into health information have become very popular [187]. One of the most common technologies in this field is non-implantable wearable biosensors. These devices can be classified as mechanical, physiological, or biochemical sensors, and they have applications ranging from gait analysis to atrial fibrillation detection and continuous blood glucose monitoring (BGM) [188]. Electrospun nanofibers are well-suited for skin-interfaced materials, as they can be designed to be thin, flexible, and breathable, with a high surface-to-volume ratio and porosity. They enable effective sensing and fast transportation of analytes such as urine, sweat, and blood [189]. Studies demonstrate that electrospun nanofiber-based wearable sensor systems can monitor a wide range of physiological signals, including cardiac activity (ECG and heartbeat measurements), respiration rate, movement, pressure, temperature, and humidity [190]. For cardiac activity measurements, conductive and piezoelectric nanofiber mats have been used, and their conformal contact with the skin has yielded better signal quality than traditional sensors [191,192]. For respiratory rate measurements, humidity sensors can be used in wearable masks, based on the principle that exhaled air has a higher moisture content than inhaled air, resulting in periodic changes in relative humidity near the sensor surface. Figure 6a shows the respiration response curves for different motion states, such as standing, walking, and running, derived from signals from electrospun nanofiber-based humidity sensors integrated into wearable face masks. The amplitude and frequency of the sensor response were correlated with respiration depth and rate, respectively [193]. For detecting finger, wrist, and limb movements, electrospun nanofiber strain sensors have demonstrated high sensitivity and show potential for use in motion-sensitive devices. Figure 6b shows the response of a strain sensor to (a1) various wrist bending angles: (b1) wrist bending to the left and right sides, (c1) response of the strain sensor to deep breathing, (d1) normal breathing actions [194].

For detecting tactile sensing and pulse monitoring, nanofiber- based pressure sensors have been used [195]. Lastly, temperature and humidity sensors were used to continuously monitor skin conditions, sweat dynamics, and temperature changes [196,197]. All these examples demonstrate the potential of electrospun nanofiber systems for use in wearable, skin-interfaced platforms for continuous physiological monitoring and personalized healthcare applications. Wearable nanofiber systems can be further advanced by integrating their data with digital health platforms via telemedicine frameworks; thus, personal health-related measurements can be transferred to clinicians when needed, or automated alarms can be generated in case of emergencies [23,198]. Moreover, this kind of telemedicine-integrated system allows clinicians to monitor patients’ health status remotely, identify early signs of adverse changes in health condition, and intervene at an earlier stage, rather than relying on hospital visits [199,200]. This active monitoring approach is particularly essential for chronic disease surveillance, post-operative follow-up, and elderly care, where subtle physiological changes may otherwise go unnoticed [201,202,203]. These applications can also support personalized healthcare analysis by collecting continuous, individual-level physiological data and detecting sudden deviations from normal patterns. To sum up, telemedicine-assisted wearable biosensor technologies can significantly improve patient monitoring, reduce barriers to accessing healthcare services, and enhance the efficiency of personalized healthcare.

### 5.4. Theranostic Nanofiber Platforms

Theranostic systems combine therapeutic and diagnostic functions in a single platform, enabling the identification of diseased cells and their simultaneous treatment [204]. Recently, electrospun nanofibers have been considered as up-and-coming theranostic systems due to their high surface area, controllable porosity, and adjustable structure. Theranostic systems are primarily explored in cancer research due to the high need for early detection, targeted treatments, and simultaneous therapy monitoring [205,206,207]. One strategy in cancer theranostics is the integration of magnetic nanoparticles with nanofibers [208]. For example, Nikolaou and colleagues produced magnetic electrospun microrods from poly(L-lactic acid) (PLLA) and poly(ethylene oxide) (PEO) polymers that are integrated with oleic-acid-coated Fe_3_O_4_ nanoparticles. They indicated that these microrods can be used for targeted lung cancer treatments by combining magnetic hyperthermia (localized heat generation induced by an alternating magnetic field) with chemotherapy [209]. Similarly, Matos and co-workers developed cellulose acetate nanofibers integrated with iron oxide nanoparticles, and the nanofibers demonstrated significant improvement in the heat generation when combined with magnetic nanoparticles for magnetic hyperthermia [210].

Another field in which electrospun nanofibers are used is photodynamic theranostics. Nanofiber systems integrated with photosensitizers (PSs) generate reactive oxygen species (ROS) upon light irradiation and can provide anticancer or antimicrobial effects while enabling optical diagnosis and response [211]. In this context, Abdelkhalek and co-workers produced cellulose acetate/polyethylene oxide (PEO) nanofibers loaded with methylene blue for photodynamic therapy mediated biofilm disruption and subsequent antibiotic release to eliminate residual bacteria [212]. These dual-action nanofibers significantly enhanced healing in an animal model of diabetic foot ulcers. In another study, Sun and co-workers produced nitric oxide (NO)-releasing membranes that utilized ROS generated during photodynamic therapy to trigger targeted NO production, thereby improving antibacterial effectiveness [213]. Thus, photodynamic theranostic systems that enable non-invasive, targeted therapy with minimal systemic side effects appear to be attractive for many biomedical applications. Collectively, theranostic nanofiber platforms integrate treatment and diagnostic functions in a single system, and their potential to combine diagnosis, simultaneous monitoring, and targeted therapy makes them promising candidates for future personalized and smart biomedical applications.

## 6. Strategic Integration of Electrospinning with 3D Printing

**Figure 7:**
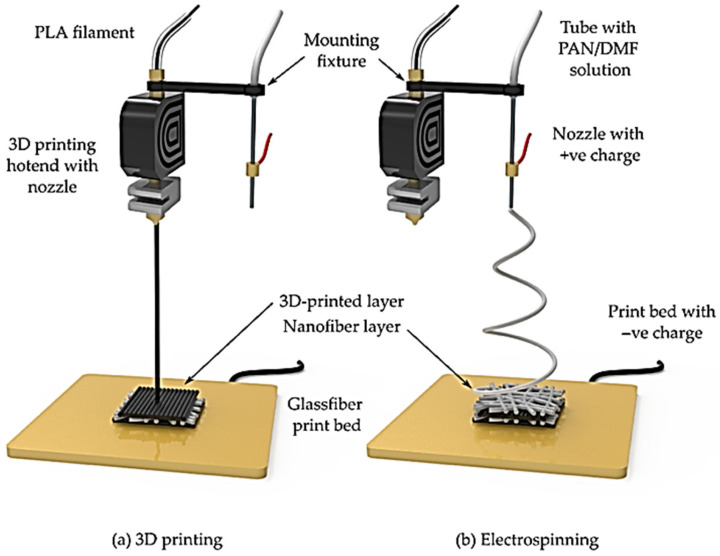
Schematic representation of the setup for producing electrospun-3D printed hybrids: (**a**) Fused Deposition Modeling 3D printing process showing the PLA filament being extruded through the nozzle onto the glass print bed, (**b**) electrospinning process showing the PAN/DMF solution spun through the needle with positive charge, creating nanofibers that are stacked onto the negatively charged print bed containing the 3D-printed layer. Reproduced from Ref. [220] under the terms of the Creative Commons Attribution License (CC BY).

Electrospun biopolymer nanofibers can mimic the structure of human ECM effectively [214]. However, relying solely on electrospinning offers limited control over the three-dimensional (3D) nanofiber structure and internal pore connectivity. To obtain specific 3D structures, 3D printing offers high control over scaffold architecture, mechanical properties, and design [27,215]. Thus, an integrative fabrication strategy that combines electrospinning with 3D printing has emerged as a robust approach to create bioactive, specifically designed, and functional scaffolds with high porosity, sufficient mechanical strength, and an ECM-mimicking structure [27,216]. This approach has been used effectively for skin, cartilage, and bone tissue engineering, in which each layer can be engineered to support required cell populations or distinct healing phases [217,218,219]. One of the most used techniques for this integration is layer-by-layer (LbL) assembly of electrospun nanofibers with 3D-printed structures. For example, Sanchaniya and colleagues combined fused deposition modeling (FDM) 3D printing with electrospinning by sequentially depositing electrospun polyacrylonitrile (PAN) nanofibers directly onto each 3D printed polylactic acid (PLA) layer in a fully automated layer-by-layer assembly (Figure 7), and they demonstrated fiber–matrix infiltration and improved mechanical performance [220].

Hybrid electrospinning/3D printing methods combine the nanofibrous structure of electrospun scaffolds with the precise macroscopic geometry provided by 3D printing. Compared to melt electro-writing and microfluidic fiber fabrication, electrospinning provides several advantages: it can produce ultrafine fibers with diameters in the nanometer scale, enabling high surface area, and mimics extracellular matrix topography that supports cell adhesion and proliferation [221]. However, electrospinning has limitations in structural precision and fiber alignment. Unlike melt electro-writing, which allows direct writing of fibers in well-defined patterns with micron-scale accuracy [222], or microfluidic fiber fabrication, which enables controlled fiber morphologies and core–shell architectures [223], conventional electrospinning produces randomly oriented fibers if additional alignment techniques such as rotating collectors or patterned electrodes are not employed. Consequently, hierarchical scaffold design combining nanofibers with defined macroscopic geometry requires hybrid strategies, such as electrospinning onto 3D-printed frameworks, to achieve both structural integrity and functional nanoscale architecture. Therefore, the choice of fabrication method depends on the intended application, balancing structural precision, fiber alignment, and biological performance.

Table 2 below lists representative recent studies on electrospun nanofiber systems for wound healing. The list is not exhaustive but represents the feasibility of conjoining biopolymers as bioactive agents in the development of nanofibers.

## 7. Challenges and Future Perspectives

Although electrospun biopolymer-based nanofibers are promising for tissue engineering, wound healing, and smart biomedical platforms, translational challenges limit their clinical use. The main challenge is scaling up the production of electrospun nanofibers while preserving their specific structural properties, such as fiber diameter distributions, porosity, and thickness [230]. Mass production generally requires needleless or multi-needle electrospinning to produce large quantities of nanofibers, and stricter control of process parameters such as humidity, temperature, polymer solubility, and electric field stability [231,232]. Thus, addressing these limitations and controlling nanofiber quality are crucial for the clinical translation of nanofibers. In addition, hybrid fabrication strategies that integrate additive manufacturing (3D printing) with electrospinning can facilitate the transition of electrospun biopolymer nanofibers from promising prototypes to routine biomedical practice. Another important consideration is the stability of natural biopolymers in aqueous environments. Many electrospun biopolymer nanofibers, such as collagen and gelatin nanofibers, require blending with synthetic polymers or crosslinking to achieve adequate mechanical strength and controlled degradation rates [233,234]. However, crosslinking can alter bioactivity, leave cytotoxic residues, and affect sterilization and shelf life [235,236]. Safe crosslinking methods and material formulations compatible with sterilization should be considered. Moreover, regulatory and clinical translation issues should be given particular consideration for bioactive agent-loaded nanofibers, theranostics, and biosensor-integrated smart scaffolds, as they are generally classified as combination products by the Food and Drug Administration (FDA) [237]. They are defined as products that combine bioactive agents with medical devices, and the advanced system must meet safety and performance requirements for both [238]. Furthermore, wearable nanofiber-based sensors should exhibit reliable sensing accuracy under real-world conditions, including during motion, exposure to sweat or other liquids, and long-term use [239,240,241]. In addition, as these devices interface with telemedicine and digital healthcare applications, safe and efficient integration with these systems, along with adherence to valid data processing standards, the provision of clinically interpretable physiological outputs, and secure data transmission, will be required [242]. Future research is expected to focus on green electrospinning processes, hybrid nanomaterials, advanced immobilization strategies, scalable fabrication techniques, and integration with wearable and AI-enabled diagnostic platforms. Overall, future perspectives for this review point toward (i) scalable manufacturing, (ii) safer and stable biopolymer chemistries, and (iii) clinically translatable multifunctional systems that integrate sensing or drug delivery with regulatory oversight and ethical considerations.

## 8. Conclusions

Electrospun biopolymer nanofibers have emerged as highly promising biomaterials for a range of biomedical applications, including tissue engineering, wound healing, and smart biomedical platforms. Their ECM-like structure, high porosity, and controllable physicochemical properties effectively support cell interactions, the controlled delivery of bioactive agents, and wound healing. Beyond their regenerative uses, recent research focuses on their use in smart, stimuli-responsive applications, biosensors, wearable devices, and theranostics. Furthermore, hybrid fabrication strategies that combine electrospinning with 3D printing allow improved structural control, mechanical strength, and functionality for nanofiber scaffolds. Recent developments also underscore the potential of integrating plant-derived bioactive compounds as functional additives into electrospun nanofibers, which offer sustainable, biologically active alternatives for advanced biomedical applications. Overall, this review highlights how integrating material design, nanofiber engineering, and multifunctional biomedical platforms can enhance the capabilities of electrospun biopolymer systems. By bringing together recent advances in fabrication strategies, functionalization approaches, and emerging biomedical applications, this work provides a comprehensive overview of the evolving role of electrospun biopolymer nanofibers in next-generation regenerative and smart healthcare technologies.

## Figures and Tables

**Table 1:** Properties of key biopolymers used in electrospun nanofiber fabrication for wound healing and tissue regeneration.

Biopolymer	Origin	Key Advantages	Key Limitations	Common Blending Partners	Ref.
Chitosan	Natural (chitin)	Antimicrobial, hemostatic, biocompatible, promotes cell proliferation	Difficult to electrospun alone; limited mechanical strength	PVA, PEO, PCL, gelatin	[118,119,120,121]
Collagen	Natural (protein)	Excellent ECM biomimicry; promotes cell adhesion/migration via RGD motifs	Weak mechanical properties; requires harsh solvents; rapid degradation	PCL, PLA, chitosan	[122,123,124]
Gelatin	Natural (collagen derivative)	Biocompatible; RGD motifs; low immunogenicity; good water retention	Poor mechanical strength; rapid aqueous dissolution; thermal instability	PLA, PCL, PLGA, PVA	[125,126,127,128,129,130]
Cellulose	Natural (polysaccharide)	High moisture retention, high structural stability	Limited bioactivity, difficult solubility for electrospinning; requires harsh solvents	PVA, PEO, gelatin, chitosan, PCL	[131,132,133]
Silk Fibroin	Natural (protein)	Exceptional mechanical strength/toughness; slow tunable degradation	Requires removal of sericin; limited bioactivity vs. ECM proteins	PEO, PCL, gelatin	[134,135]
Alginate	Natural (polysaccharide)	High water absorption (15–20×); promotes moist environment; autolytic debridement	Difficult to electrospun alone; poor mechanical properties	PEO, PVA, chitosan, collagen	[131,136]
Hyaluronic Acid	Natural (GAG)	Critical ECM component; exceptional hydration; regulates inflammation and angiogenesis	Difficult to electrospun alone; rapid enzymatic degradation	PVA, PCL, gelatin, PEO	[137,138]
PLA	Synthetic (polyester)	FDA-approved; good mechanical properties; controllable degradation (6–24 months)	Hydrophobic; lacks cell recognition sites; acidic degradation products	Gelatin, chitosan, collagen	[139,140]
PCL	Synthetic (polyester)	FDA-approved; excellent flexibility; slow degradation (2–4 years); easy to electrospun	Hydrophobic; poor cell adhesion without modification	Chitosan, collagen, gelatin, HA	[141,142]
PVA	Synthetic (vinyl polymer)	Water-soluble; non-toxic; excellent co-spinning agent; aqueous processing	Rapid dissolution without crosslinking; limited standalone mechanical properties	Chitosan, alginate, HA, proteins	[143]

**Table 2:** Representative Recent Studies on Electrospun Nanofiber Systems for Wound Healing.

Nanofiber Composition	Bioactive Agent(s)	Key Functionalities	Key Findings	Ref.
PCL/Chitosan	Silver nanoparticles	Antimicrobial, hemostatic	Broad-spectrum activity against *S. aureus* and *P. aeruginosa*; ~95% wound closure in 14 days in the rat model	[224]
Gelatin/PLA	Curcumin	Anti-inflammatory, antioxidant	Sustained curcumin release over 14 days; reduced TNF-α and IL-6 levels; enhanced fibroblast proliferation	[225]
PCL/Collagen (core–shell)	VEGF + bFGF (dual release)	Pro-angiogenic, regenerative	Sequential growth factor release; 3-fold increase in blood vessel density; complete wound closure by day 12	[226]
Silk Fibroin/PEO	EGF	Re-epithelialization, ECM mimicry	Sustained EGF release for 21 days; 2-fold increase in keratinocyte migration rate; thicker neo-epidermis	[227]
Chitosan/PVA	ZnO nanoparticles + aloe vera	Antimicrobial, anti-inflammatory, hydrating	Synergistic antibacterial effect; reduced inflammation; ~90% wound closure in 10 days	[224]
PCL/Gelatin	Dexamethasone (pH-responsive shell)	Smart anti-inflammatory release	Drug release triggered at alkaline pH (infection); 60% reduction in inflammatory infiltrate	[228]
PVA/Alginate	Honey + tetracycline	Antimicrobial, autolytic debridement	Dual-action dressing; effective against biofilm-forming bacteria; enhanced moist wound environment	[166]
PLGA/HA	PDGF	Fibroblast recruitment, ECM synthesis	Controlled PDGF release for 28 days; 40% increase in collagen type I deposition; improved scar quality	[229]

## Data Availability

No new data were created or analyzed in this study. Data sharing is not applicable to this article.

## Abbreviations

The following abbreviations are used in this manuscript:

BDNF	Brain-Derived Neurotrophic Factor
BGM	Blood glucose monitoring
CNS	Central nervous system
CQD	Carbon quantum dot
ECG	Electrocardiography
ECM	Extracellular matrix
FDM	Fused deposition modeling
GCE	Glassy carbon electrodes
GNR	Gold nanorods
HA	Hyaluronic acid
hTSPCs	human tendon progenitor cells
LbL	Layer by Layer
NGF	Nerve Growth Factor
NIR	Near-Infrared
NO	Nitric oxide
NT	Neurotrophin
PAN	Polyacrylonitrile
PANi	Polyaniline
PCL	Poly(ε-caprolactone)
PEO	Poly(ethylene oxide)
PLA	Polylactic acid
PLGA	Poly(lactic-co-glycolic) acid
PLLA	Poly(L-lactic acid
PNIPAM	Poly(N-iso-propylacrylamide)
PNS	Peripheral nervous system
PPY	Polypyrrole
PVA	Polyvinyl alcohol
RGD	Arginine-Glycine-Aspartic Acid
ROS	Reactive oxygen species
SF	Silk Fibroin
